# A systematic review and meta‐analysis of treatments for rapid cycling bipolar disorder

**DOI:** 10.1111/acps.13471

**Published:** 2022-07-20

**Authors:** Rebecca Strawbridge, Suman Kurana, Jess Kerr‐Gaffney, Sameer Jauhar, Kenneth R. Kaufman, Nefize Yalin, Allan H. Young

**Affiliations:** ^1^ Department of Psychological Medicine Institute of Psychiatry, Psychology & Neuroscience, King's College London London UK; ^2^ South London & Maudsley NHS Foundation Trust London UK; ^3^ Department of Psychiatry Rutgers Robert Wood Johnson Medical School New Brunswick New Jersey USA

**Keywords:** bipolar disorders, meta‐analysis, rapid cycling, systematic review, treatment

## Abstract

**Objectives:**

Rapid cycling is a common and disabling phenomenon in individuals with bipolar disorders. In the absence of a recent literature examination, this systematic review and meta‐analysis aimed to synthesise the evidence of efficacy, acceptability and tolerability of treatments for individuals with rapid cycling bipolar disorder (RCBD).

**Method:**

A systematic search was conducted to identify randomised controlled trials assigning participants with RCBD to pharmacological and/or non‐pharmacological interventions. Study inclusion and data extraction were undertaken by two reviewers independently. The primary outcome was continuous within‐subject RCBD illness severity before and after treatment. Pre‐post random effects meta‐analyses were conducted for each outcome/intervention arm studied, generating a standardised effect size (hedge's *g*) and 95% confidence interval (CI).

**Results:**

A total of 34 articles describing 30 studies were included. A total of 16 separate pharmacological treatments were examined in contrast to 1 psychological therapy study. Only quetiapine and lamotrigine were assessed in >5 studies. By assessing 95% CI overlap of within‐subject efficacy effects compared to placebo, the only interventions suggesting significant depression benefits (placebo *g =* 0.60) were olanzapine (with/without fluoxetine; *g =* 1.01), citalopram (*g =* 1.10) and venlafaxine (*g =* 2.48). For mania, benefits were indicated for quetiapine (*g =* 1.01), olanzapine (*g =* 1.19) and aripiprazole (*g =* 1.09), versus placebo (*g =* 0.33). Most of these effect sizes were from only one trial per treatment. Heterogeneity between studies was variable, and 20% were rated to have a high risk of bias.

**Conclusions:**

While many interventions appeared efficacious, there was a lack of robust evidence for most treatments. Given the limited and heterogeneous evidence base, the optimal treatment strategies for people with RCBD are yet to be established.

**Summations**

- The only treatment class with >1 study examining mania was antipsychotics, which appeared more beneficial than placebo comparators. Quetiapine was the best studied treatment and this appeared consistently beneficial both of depressive and manic poles.
- Mood stabilisers had higher effect size (ES) for depression and lower for global impression, compared to antipsychotics. Within this class, lithium had the highest ES across both outcomes.
- Antidepressants showed higher ES's for both depression and global impression outcomes compared to the other treatment classes. Venlafaxine had the highest ES but most safety concerns, while SSRIs indicated moderate efficacy and safety, and bupropion reported narratively to be both effective and safe.

**Limitations**

- Few studies, particularly for hormone and non‐pharmacological therapies. We propose, however, that the current findings remain clinically relevant, especially with rapid cycling being a particularly difficult‐to‐treat form of bipolar disorder and given the current absence of treatment guidelines for this population
- Tolerability/harms were not comprehensively assessed (including affective switch), despite being important. This was due to a lack of reporting consistent outcomes and data reporting specifically on rapid cycling patients.
- Within‐subjects meta‐analyses do not allow a statistical comparison between active/control interventions, and have been criticised for effect sizes incorporating non‐specific factors such as natural recovery. We maintain that despite these legitimate arguments, pre‐post estimates have good clinical face validity and maximise includable data, in this case permitting a quantitative synthesis which otherwise may not have been viable.

## 1 INTRODUCTION

The extensive disability burden of bipolar disorders (BD) is predominantly attributable to the high lifetime recurrence of affective episodes.^1^ People with rapid cycling bipolar disorder (RCBD) experience particularly high rates of recurrence.^1^ The Diagnostic and Statistical Manual of Mental Disorders 5th edition (DSM‐5) criteria for the RCBD specifier requires occurrence of ≥4 distinct episodes (meeting criteria for full mania, hypomania, or major depression), within a 12‐month period. These affective episodes are permitted in any sequence and combination, but have to be demarcated by partial or full remission of at least 2 months or comprise switch to a mood episode of the opposite polarity.^2^ This definition has persisted since its inception.^3^

The increased frequency of affective episodes for people with RCBD is stark; they experience approximately a sevenfold greater mean number of full (hypo)manic episodes and twofold greater depressive episodes in comparison to those with (non‐RC) BD.^4^ Estimates of the 12‐month prevalence of RCBD are variable but lifetime prevalence is between 26% and 43% of those with BD, and these individuals experience a substantial illness burden including a higher prevalence of various physical health conditions, episodes with mixed features, comorbid substance use disorders, suicidality, poorer psychosocial functioning and greater episode burden.^5^

As such, RC contributes significantly to overall BD illness burden and constitutes a critical, unsolved clinical challenge.^6^ Treatment guidelines for BD do not recommend any specific treatment for RCBD,^7^ despite reports of an unfavourable response to current interventions, suggesting more specific guidance would be valuable.^8^ This lack of recommendations for RCBD likely reflects a lack of attention in clinical trials (which often exclude people meeting this specifier) and consequent lack of evidence‐based consensus on optimal treatment strategies.^5^

A systematic review collating evidence on interventions for RCBD was conducted over a decade ago (2011). This review considered only pharmacological therapies and identified few studies, precluding a quantitative synthesis of the literature and its findings were therefore inconclusive.^9^

## 2 OBJECTIVES

As the first meta‐analysis of treatments for RCBD, this review aimed to synthesise the evidence for pharmacological and non‐pharmacological treatments for people with this illness, by identifying all relevant randomised trials, pooling and comparing within‐subjects effect sizes (ES) and confidence intervals (CI) across interventions. The primary objective was to estimate efficacy parameters for each eligible treatment and control arms. Our secondary objectives were to indicate data on their acceptability (i.e., discontinuation) and tolerability (i.e., adverse effects) as well as binary efficacy measures (e.g., % response).

## 3 METHODS

This systematic review adhered to the preferred reporting items for systematic reviews and meta‐analyses (PRISMA) guidelines.^10^ The protocol was pre‐registered to the international prospective register of systematic reviews (pre‐registered protocol ID: CRD42021231096 (PROSPERO, 2021).

### 3.1 Eligibility criteria

To be considered eligible for inclusion, studies had to be randomised controlled trials (RCTs), including crossover trials (where the first treatment phase would be focused on in analyses) where ≥10 eligible participants were randomised. Eligible participants had to be adults (aged ≥18), of any gender, with a BD diagnosis that currently fulfilled standardised criteria for RC. Participants could be in any mood state at baseline. Interventions trialled were considered eligible for inclusion if they were recommended by any of the referenced clinical guidelines more broadly for BD (pharmacological and non‐pharmacological).^6^, ^7^, ^11^, ^12^, ^13^ Eligible comparator arms included pill placebo, another pharmacological or non‐pharmacological intervention, waiting list or treatment as usual. Studies needed to have reported on outcomes related to efficacy, or clinical improvement.

### 3.2 Systematic search and information sources

The electronic databases PubMed, PsycInfo and EMBASE were searched (all dates from inception to 14 February 2021) in addition to hand‐searched citation lists from notable papers, recent relevant reviews and included articles. Relevant reviews for handsearching were sourced from the primary search and from the Cochrane Library. The Cochrane library was also re‐searched post‐hoc (June 2022) with no additional eligible articles identified. The following subject headings or text word terms were used for the electronic database search, which was run for all databases concurrently using Ovid: (bipolar OR manic‐depress* OR manic OR mania) AND (rapid OR ultradian* OR cycling OR rapid‐cycling) AND (randomi* OR RCT or trial* OR treatment OR intervention OR psychological OR mood stabili* OR antipsychotic* OR antidepressant*). No language restriction was made.

### 3.3 Study selection and data collection process

Rayyan open‐source review management software^14^ was utilised to assist the review process. The titles and abstracts of all search results were independently evaluated by two reviewers (SK and RS or KRK). The review objectives were not concealed from any reviewers. Both reviewers independently screened each article against stipulated eligibility requirements (blinded from the others' selections). Subsequently, the two reviewers reached consensus on eligible studies with the support of an additional reviewer (AHY). The same process was followed for the full‐text screening of all articles that were determined as being potentially eligible. Following inclusion, data extraction was conducted by SK and JKG independently as above (with discrepancies resolved through consensus with RS).

The following information was extracted from all included articles: methodological and publication details (e.g., setting, assessment of baseline characteristics, treatment duration, interventions, doses), results (participant characteristics, data related to primary outcomes of efficacy, secondary outcomes of response/remission/relapse, tolerability and acceptability) in addition to a risk of bias assessment. Authors of articles that could not be included due to lack of RCBD participant data were contacted to request this.

The Risk of Bias (RoB) assessment comprised an adapted version of The Cochrane RoB2 tool.^15^ Given the heterogeneity of study designs and review focuses, RoB tools tailored to specific designs can add value,^16^ and our adaptation examined the same domains with the addition of potential allegiance effects (in this case, indications of for‐profit bias). After agreement on individual criterion ratings, each study was assigned an overall RoB rating of low, moderate or high.^17^

### 3.4 Outcome measures

#### 3.4.1 Primary outcome measure(s)

The primary outcome was treatment efficacy as assessed using continuous measures assessed before and after intervention period. These outcomes were categorised as measures of:

- Global impression or severity of BD illness (using e.g., a clinical global impression [CGI] measure^18^ or multi‐faceted symptom scale covering several affective domains (e.g., depression, anxiety, mania, psychosis, functional status) and therefore representing a more encompassing outcome^19^);
- Depression severity using a specific depressive symptom rating scale (e.g., Montgomery‐Asberg depression rating scale [MADRS]^20^ or Hamilton depression rating scale [HAMD]^21^);
- Mania severity using a specific manic symptom rating scale (e.g., Young Mania rating scale [YMRS]^22^);
- Other assessments usually indicating time to improvement (response) or time to deterioration (relapse/medication change). Where multiple measures per category were reported, validated clinician‐rated measures were prioritised over patient‐rated measures. In cases where several post‐treatment timepoints were reported, the last post‐treatment time point was selected for analysis.

#### 3.4.2 Secondary outcome measure(s)

Where available, the following additional outcomes were assessed: 1) Binary assessments pertaining to efficacy (usually rate of response, remission or relapse present at treatment endpoint); 2) A measure of tolerability (e.g., adverse event or side effects data); 3) measure of acceptability (e.g., study dropout or intervention discontinuation). Further detail related to the extraction and presentation of secondary outcome data can be found in Supplement 1.

### 3.5 Analysis of outcomes

#### 3.5.1 Primary outcome

Where sufficient data (for each of the four categories of efficacy outcome) were available, continuous measures before and after treatment (as described above) were entered into Comprehensive Meta‐Analysis software calculating a hedges' *g* standardised mean difference, otherwise termed effect size (ES). Utilising a random‐effects model, a meta‐analysis computed a pooled ES with 95% confidence intervals (CI). I^2^ statistic was computed to denote heterogeneity of effects between studies within each analysis. Heterogeneity was considered significant where I^2^ > 60%.^23^ Sources of heterogeneity were examined using sensitivity or subgroup analyses (as described below). These pooled within‐subjects meta‐analyses were planned for each efficacy category, both for individual treatments studied and classes of interventions (e.g., mood stabiliser, antipsychotic, antidepressant, psychological, placebo, usual care). Only participants who were depressed at baseline were included in analysis of depression outcomes, only manic participants included in mania outcomes, and euthymic participants were not included in any of the first three category (global severity, depression, mania) comparisons. Analyses pertaining to the fourth category were undertaken based on data availability.

Statistical significance of between‐arm comparisons cannot be determined directly from within‐subject meta‐analyses, but indicative significance can be estimated by comparing instances where 95% confidence intervals for each comparison do not overlap with others.

#### 3.5.2 Subgroup and sensitivity analyses

A range of possible subgroup analyses were noted in the protocol, planned for consideration depending on the quantity and characteristics of data available. Prior to analyses we highlighted studies that should be considered for removal after sensitivity analysis if they met any of the following criteria: 1) high RoB rating, 2) where analyses may have been affected by some participants without RCBD being included, or 3) where baseline severity related to the outcome was mild or patients were described as being in partial remission. Sensitivity analysis then examined whether exclusion of these studies reduced heterogeneity estimates of meta‐analyses; where they did, the study was excluded from main analyses and where not the study was included. Study duration was explored in subgroups, separating studies of <6, 6–52 weeks and >52 weeks. These particular durations were used, as it was felt that <6 weeks represented a potentially inadequate length of even acute treatment, and conversely that 52 weeks is assessing a broade RCBD response (with 52 weeks being the duration needed to determine whether an individual meets current criteria for rapid cycling). Subgroups that were described in the review protocol as potentially to be investigated (pending data availability), but were not, were: monotherapy versus combination therapies (due to insufficient variability), BD type I versus type II participants (due to limited datapoints prior to subgroup analyses, per comparison) and studies where participants were identified as having an organic neuropsychiatric condition (none applicable).

#### 3.5.3 Secondary outcomes

The aforementioned secondary outcomes were planned to be assessed narratively through presentation in tables alongside text summaries.

### 3.6 Amendments after protocol registration

One change to the protocol was considered necessary subsequent to protocol registration (specifically, at full text screening phase): A number of studies did not report separate results between included participants with/without rapid cycling. It was decided to include those where RC participants comprised the majority (≥75%) of the sample and/or where the authors reported there to be no significant effect of RC status on outcomes (i.e., no difference in efficacy results between individuals with versus without rapid cycling). The primary reason for this is the clear benefits of incorporating all possible evidence from RCTs indicating treatment efficacy for this population.

## 4 RESULTS

### 4.1 Systematic search results

**FIGURE 1:**
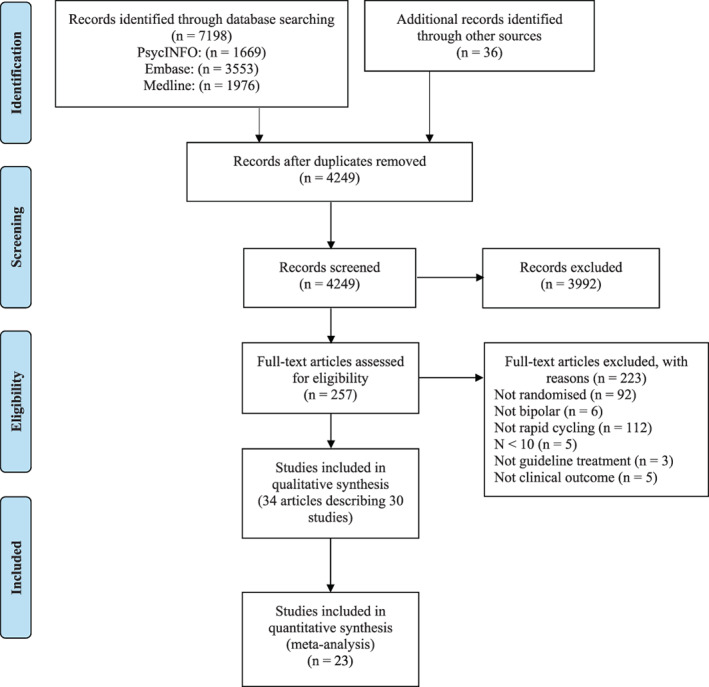
PRISMA flow diagram of the study selection process.

As illustrated in the PRISMA flowchart (Figure 1), the systematic search generated 7234 records (4249 after removing duplicates). Screening revealed 257 potentially eligible articles whose full texts were reviewed. Notably, several articles reported including participants with RCBD in an otherwise‐eligible study, but did not report outcomes specifically on these patients. Authors of such studies were contacted for potentially relevant unpublished data. Two eligible studies were identified and included through this process.^24^, ^25^ Overall, 34 articles describing 30 studies were eligible for inclusion. A record of studies that were reported in multiple articles, and multiple studies that were reported in a single article can be found in Supplement 2.

### 4.2 Characteristics of included studies

The study characteristics of all included trials are presented in Table 1 (and further supplementary information in Supplement 3). Across studies, 2266 RC participants were randomised. 21 (70%) trials were conducted solely in North America, 6 (20%) were conducted across multiple continents, 2 (7%) were conducted within Europe and one was not reported. The mean study size was 78 (*SD* = 65, range 10–293). The duration of interventions ranged from 3 weeks (quetiapine^26^) to 100 weeks (aripiprazole^27^); median duration 14 weeks. One study was a crossover trial,^28^ and only one examined a non‐pharmacological intervention (cognitive psychoeducational therapy [CPT], compared with bibliotherapy).^24^

**TABLE 1:** Characteristics of included studies.

References	Intervention	Setting	Population	Minimum criteria for RC	Maximum criteria for RC	% RCBD	Current Episode
*Antipsychotics*							
Tohen et al. 2006	Olanzapine vs. Placebo	IN/OP	BD‐I	≥4 episodes in last year	n/a	50%	Euthymia
Baldessarini et al. 2003^a^ ^,^ ^b^	Olanzapine vs. Placebo	VAR	BD‐I	DSM‐IV	n/a	35%	Mania, mixed
Sanger et al. 2003^a^		IN/OP				100%	Mania, mixed
Suppes et al. 2014	Quetiapine vs. Placebo	OP	BD‐I/BD‐II	≥4 episodes in last year	≥8 episodes in last year	27%	Depression
Cutler et al. 2011	Quetiapine vs. Placebo	IN/OP	BD‐I	DSM‐IV	≥8 episodes in last year	31%	Mania, mixed
Vieta et al. 2007^a^	Quetiapine vs. Placebo	OP	BD‐I/BD‐II	≥4 episodes in last year	n/a	21%	Depression
Thase et al. 2006	Quetiapine vs. Placebo	OP	BD‐I/BD‐II	DSM‐IV	n/a	31%	Depression
Muzina et al. 2008^a^	Aripiprazole vs. Placebo	OP	BD‐I	DSM‐IV	n/a	100%	Euthymia
Suppes et al. 2008b^a^ ^,^ ^b^	Aripiprazole vs. Placebo	IN/OP	BD‐I	DSM‐IV	n/a	20%	Mania/mixed
Bobo et al. 2011	Risperidone LAI vs. TAU	OP	BD‐I/BD‐II	≥4 episodes in last year	n/a	100%	Depression, (hypo)mania
*Mood stabilisers*							
Kemp et al. 2012	Lamotrigine vs. Placebo	OP	BD‐I/BD‐II	DSM‐IV	n/a	100%	Depression
Wang et al. 2010	Lamotrigine vs. Placebo	OP	BD‐I/BD‐II	DSM‐IV	n/a	100%	Depression, mixed, (hypo)mania
Suppes et al. 2008	Lamotrigine vs. Lithium	OP	BD‐II	DSM‐IV	n/a	72%	Depression
Calabrese et al. 2000^a^	Lamotrigine vs. Placebo	OP	BD‐I/BD‐II	DSM‐IV	n/a	100%	(partially) remitted
Goldsmith et al. 2003	Lamotrigine vs. Placebo	OP	BD‐I/BD‐II	DSM‐IV	>20 episodes in last year	100%	(partially) remitted
Walden et al. 2000	Lithium vs. Lamotrigine	OP	BD‐I	≥4 episodes in last year	>12 episodes in last year	100%	Mania
*Antidepressants*							
Ghaemi et al. 2021	Citalopram vs. Placebo	OP	BD‐I/BD‐II	DSM‐IV	n/a	28%	Depression
Parker et al. 2006	Escitalopram vs. Placebo	CO	BD‐II	≥1 episode per month	n/a	100%	Depression, euthymia
Post et al. 2006^a^	Venlafaxine vs. Bupropion vs. Sertraline	OP	BD‐I/II/NOS	DSM‐IV	n/a	27%	Depression
*Other classes*							
Walshaw et al. 2018	Levothyroxine vs. T3 vs. Placebo	OP	BD‐I/BD‐II	≥4 episodes in last year	n/a	100%	Depression, (hypo)mania
Keck et al. 2006	Ethyl‐EPA vs. Placebo	OP	BD‐I/II/NOS	DSM‐IV	n/a	51%	Depression, (hypo)mania
Lenz et al. 2016	CPT vs. Bibliotherapy	OP/IN	BD‐I/BD‐II ^c^	≥8 episodes in last 2 years	n/a	16%	(partially) remitted
*Multiple classes*							
Amsterdam et al. 2017^a^	Venlafaxine vs. Lithium	OP	BD‐II	DSM‐IV & ≥4 episodes per year average	n/a	47%	Depression
Amsterdam et al. 2013	Fluoxetine vs. Lithium vs. Placebo	OP	BD‐II	≥4 episodes per year average	n/a	31%	Euthymia
Amsterdam et al. 2009	Venlafaxine vs. Lithium	OP	BD‐II	≥4 episodes per year average	n/a	32%	Depression
Suppes et al. 2005^a^	Olanzapine vs. Divalproex	IN/OP	BD‐I/BD‐II	≥4 episodes in last year	n/a	58%	Mania, mixed
Tohen et al. 2003	Olanzapine vs. Placebo vs. OFC	IN/OP	BD‐I	≥4 episodes in last year	n/a	37%	Depression
Langosch et al. 2008	Quetiapine vs. Na Val	NR	BD‐I/BDII	DSM‐IV	n/a	100%	(partially) remitted
McElroy et al. 2010	Quetiapine vs. Paroxetine vs. Placebo	NR	BD‐I/BDII	≥4 episodes in last year	>8 episodes in last year	18%	Depression

Abbreviations: BD‐I, bipolar disorder type I; BD‐II, type II; CO, community; CPT, cognitive psychoeducational therapy; DSM‐IV, Diagnostic & Statistical Manual of Mental Disorders, 4th edition; ethyl‐EPA, ethyl‐eicosapentanoate; IN, inpatient; LAI, long‐acting injectable; Na Val, sodium valproate; NA, not applicable; NOS, not otherwise specified; NR, not reported; OFC, olanzapine fluoxetine combination; OP, outpatient; OTH, other; RC, rapid cycling; RCBD, rapid cycling bipolar disorders; T_3_, triiodothyronine; TAU, treatment as usual.

^a^ Secondary analyses/multiple articles per study (see Supplement 2).

^b^ Data pooled from 2 trials (each). NB for Baldessarini/Sangar et al., Baldessarini (and other secondary articles; see Supplement 2) pools two trials, while Sangar et al. includes data from one of these trials; the overlap of data here is indicated via apparent merging of rows in the table.

^c^ ICD rather than DSM criteria.

### 4.3 Risk of bias assessment

Ten trials were judged to have a low RoB, 14 moderate and 4 high RoB^29^, ^30^, ^31^, ^32^ (although two of the latter were pooled from two identical RoB studies); thus of 30 total studies, 6 (20%) had a high RoB. There was frequently uncertainty over randomisation robustness and whether analyses deviated from protocols (in the presence of primarily older studies not reporting these details). Few studies (<15%) were rated to have a high RoB in domains of blinding, intention‐to‐treat analyses and appropriateness of outcomes examined. Many trials (>50%) compared participants with differing characteristics at baseline between groups (often signalling inadequate sample sizes randomised), and many had a potential allegiance bias (e.g., being funded by the respective pharmaceutical manufacturer) (See Supplement 4).

### 4.4 Characteristics of participants

The majority of studies included participants with current RCBD according to the DSM specifier, although two required participants to have a lifetime average of ≥4 episode per year,^33^, ^34^ one required ≥1 episode per month,^28^ and one required ≥8 episodes within a 2 year period (broadly equivalent to a current RC).^24^ 5 studies included a maximum threshold, excluding participants with more than 8,^26^, ^35^, ^36^ 12,^37^ or 20^38^ episodes in the preceding year. A total of 16 studies recruited participants with different subtypes of BD, 9 only with BD type‐I and 5 only with BD type‐II. Participants were in a variety of mood states at baseline (see Table 1). Characteristics of the studies' interventions are presented in Table 2.

**TABLE 2:** Characteristics of treatments examined.

References	Intervention	*N*	Dose	Duration (weeks)	Tolerability	Discontinuation rate (acceptability)	Efficacy comparison (RC vs. non‐RC)
*Antipsychotics*							
Tohen et al., 2006	Olanzapine Placebo	119 60	5–20 mg	48	8% discontinued due to AE^e^ 0% discontinued due to AE^e^	32%^e^ 13%^e^	NS
Baldessarini et al. 2003^a^	Olanzapine Placebo	44 46	M: 15 mg —	3–4	57% of RC experienced SAE NR	38%^e^ (RC 57% in 12 m) 62%^e^	Mostly NS. RC > NRC some ST; NRC > RC some LT^d^
Sanger et al. 2003^a^	Olanzapine Placebo	19 26	5–20 mg —	3	0% discontinued due to AE 0% discontinued due to AE	21% 54%	n/a
Suppes et al. 2014	Quetiapine Placebo	36 38	300 mg —	8	88% experienced an AE^e^ 67% experienced an AE^e^	38%^e^ 31%^e^	NS
Cutler et al. 2011	Quetiapine Placebo	45 52	M: 604 mg —	3	3% discontinued due to AE^e^ 8% discontinued due to AE^e^	28%^e^ 28%^e^	NRC > RC mania interaction
Vieta et al. 2007^a^	Quetiapine Placebo	42/31 35	300/600 mg —	8	15/24% discontinued due to AE 8% discontinued due to AE	36/48% 38%	NS
Thase et al. 2006	Quetiapine Placebo	44/46 53	300/600 mg —	8	8/11% discontinued due to AE^e^ 1% discontinued due to AE^e^	41/47%^e^ 35%^e^	NS
Muzina et al. 2008^a^	Aripiprazole Placebo	14 14	M: 24 mg —	100	7% experienced an AE 0% experienced an AE	79% 100%	n/a
Suppes et al. 2008b^a^	Aripiprazole Placebo	52 51	M: 28 mg —	3	10% discontinued due to AE^e^ 9% discontinued due to AE^e^	52%^e^ 63%^e^	NS
Bobo et al. 2011	Risperidone LAI TAU	20 25	M: 27 mg Optimised	52	MC: 20% basal ganglia, 15% sedation, 10% + weight MC: 24% basal ganglia, 16% sedation, 12% + weight	20% 24%	n/a
*Mood stabilisers*							
Kemp et al. 2012	Lamotrigine Placebo	23 26	150‐200 mg	12	4% ‐ pruritis/benign rash 4% ‐ pruritis	17% 15%	n/a
Wang et al. 2010	Lamotrigine Placebo	18 18	150‐200 mg	12	MC: 22% tremor, 11% nausea, 6% headache MC: 39% tremor, 33% headache, 11% diarrhoea	55% 55%	n/a
Suppes et al. 2008^b^	Lamotrigine Lithium	34 34	M: 250 mg M: 1200 mg	16	M(*SD*): 4.2 ± 3.2^e^ M(*SD*): 9.2 ± 6.4^e^	49%^e^ 61%^e^	NS
Calabrese et al. 2000^a^	Lamotrigine Placebo	90 87	M: 288 mg —	26	67% experienced an AE 68% experienced an AE	59% 74%	n/a
Goldsmith et al. 2003	Lamotrigine Placebo	66 68	50‐400 mg	32	85% experienced an AE 78% experienced an AE	40% 42%	n/a
Walden et al. 2000	Lithium Lamotrigine	7 7	M: 0.84 mmol/L M: 425 mg	52	43% weight gain, 29% slight tremor 43% dizziness, 29% headache	0% 0%	n/a
*Antidepressants*							
Ghaemi et al. 2021	Citalopram Placebo	14 19	M: 27 mg —	52	35% experienced an AE^e^ 37% experienced an AE^e^	77%^e^ 80%^e^	NRC > RC mania interaction
Parker et al. 2006	Escitalopram Placebo	6 4 ^c^	10 mg —	26	0% discontinued due to AE 0% discontinued due to AE	0% 0%	n/a
Post et al. 2006^a^	Bupropion Sertraline Venlafaxine	21 12 14	M: 286 mg M: 195 mg M: 192 mg	10	0% discontinued due to an AE^e^ 7% discontinued due to an AE^e^ 3% discontinued due to an AE^e^	31%^e^ 41%^e^ 45%^e^	RC bupropion > venlafaxine mania/NRC treatment NS
*Other classes*							
Walshaw et al. 2018	Levothyroxine T3 Placebo	13 10 9	= > index 4.5–7.5 = > serum 0.65–1.36 —	12–48	0% discontinued due to an AE 10% discontinued due to an AE 0% discontinued due to an AE	19% 0% 0%	n/a
Keck et al. 2006	Ethyl‐EPA Placebo	31 28	6 g	17	5% experienced an SAE^e^ 5% experienced an SAE^e^	54%^e^	NR
Lenz et al. 2016	CPT Bibliotherapy	7 9	14 × 90‐min weekly 3 × 90‐min group	14 (<14 m FU)	NR	10% 23%	NR
Multiple Classes							
Amsterdam et al. 2017^a^	Venlafaxine Lithium	17 7	37.5‐375 mg 300‐1200 mg	12	NR	59% 22%	NS
Amsterdam et al. 2013	Fluoxetine Lithium Placebo	8 9 8	10‐40 mg 300‐1200 mg —	50	0% discontinued due to AE 0% discontinued due to AE 13% discontinued due to AE	50% 89% 88%	NS
Amsterdam et al. 2009	Venlafaxine Lithium	12 15	37.5‐ 375 mg 300‐2100 mg	12	16% discontinued due to AE	40%^e^	RC > NRC across arms
Suppes et al. 2005^a^	Olanzapine Divalproex	76 68	M: 16 mg M: 1530 mg	47	MC: 18% + weight, 20% rhinitis, 7% edema MC: 17% + weight	85%^e^ 84%^e^	NRC > RC across arms
Tohen et al. 2003	Olanzapine Placebo OFC	132 127 34	5‐20 mg ‐ 6 + 25/6 + 50/12 + 50 mg	8	9% discontinued due to AE^e^ 5% discontinued due to AE^e^ 2% discontinued due to AE^e^	52%^e^ 62%^e^ 36%^e^	NRC > RC depression interaction (not stat. tested)
Langosch et al. 2008	Quetiapine Na Valproate	21 16	M: 465 mg M: 1340 mg	52	86% experienced an AE 63% experienced an AE	63% 60%	n/a
McElroy et al. 2010	Quetiapine Paroxetine Placebo	81 24 24	300/600 mg 20 mg —	8	11% discontinued due to AE^e^ 13% discontinued due to AE^e^ 8% discontinued due to AE^e^	35% 38% 40%	NRC quetiapine > placebo/RC NS

Abbreviations: =>, titrated to; AE, adverse event; CPT, cognitive psychoeducational therapy; ethyl‐EPA, ethyl‐eicosapentanoate; FU, follow‐up; LAI, long‐acting injectable; LT, long‐term; M, mean/median; MC, most common; *n*, number of patients; NR, not reported; NRC, non‐rapid cycling; NS, not significant; RC, rapid cycling; SAE, serious adverse event; ST, short‐term; T_3_, triiodothyronine; TAU, treatment as usual; URI, upper respiratory infection.

^a^ Multiple papers per study, & in some cases multiple studies reported per article (see Supplement 2).

^b^ Data extracted from the whole study sample as most participants had RC and/or where no difference between RC and NRC was reported.

^c^ Reports data across all treatment groups.

^d^ Vieta et al. reported similar mania improvements in the short‐term between RC and non‐RC participants, but a higher proportion of RC patients responded in the 3–4 week blinded trial, and responded more quickly than non‐RC patients (across olanzapine and placebo arms). However, in the long‐term (1 year open label olanzapine treatment), although parity of several outcomes was reported between these subgroups, better outcomes were identified for non‐RC versus RC participants in: remission/recovery rates, time to recovery, number of episodes, rehospitalisation and suicide rates, and tolerability difficulties.

^e^ Describes data covering the overall study group as separate results for RC participants were not reported.

### 4.5 Primary outcome pre‐post meta‐analyses

**FIGURE 2:**
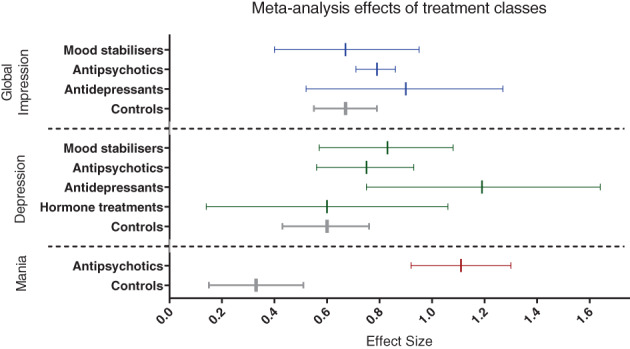
Meta‐analysis effects of treatment classes. Forest plot displaying meta‐analysis comparisons (class level) pooled from >1 arms. Pre‐post effect size (Hedges' g) and 95% confidence intervals are shown. Blue colour represents treatment effects on global impression; green on depression; red on mania; grey colour displays control arms within each outcome category.

The pre‐post meta‐analysis efficacy findings are described below and in Table 3, with Figure 2 depicting the class‐level effect sizes for each outcome. Further information about studies' assessment of various efficacy outcomes can be found alongside secondary (binary) efficacy results in Supplement 5. Details of the studies removed in sensitivity analyses and rationale for this are tabled in Supplement 6. Results for “other” efficacy outcomes (such as time to relapse or improvement) were assessed in far fewer studies than either global impression, depression or mania, and are summarised in Supplement 7. The effect of treatment duration on meta‐analysis outcomes was assessed in comparisons of ≥3 studies, described below where there was a duration effect, and are tabled in full in Supplement 8. Below results are given for class‐level analyses, and individual treatments where examined in >1 trial.

**TABLE 3:** Results of meta‐analyses assessing primary outcome.

Treatment	Global impression						Depression						Mania					
	*k*	*N*	ES	*SE*	95% CI	I^2^	*k*	*N*	ES	*SE*	95% CI	I^2^	*k*	*N*	ES	*SE*	95% CI	I^2^
Antipsychotics	6	900	0.79	0.04	0.71–0.86	0%	10	1131	0.75	0.09	0.56–0.93	69%^a^	3	287	1.11	0.01	0.92–1.30	0%^d^
Quetiapine	4	804	0.79	0.04	0.71–0.87	0%	5	855	0.76	0.09	0.59–0.92	37%^a^	1	159	1.01	0.18	0.65–1.37	—
Olanzapine/OFC	1	76	0.87	0.14	0.60–1.13	—^b^	3	242	1.01	0.08	0.86–1.17	0%	1	76	1.19	0.15	0.90–1.49	—^d^
Aripiprazole	—	—	—	—	—	—	1	14	0.06	0.27	−0.47 to 0.58	—	1	52	1.09	0.18	0.75–1.43	—
Risperidone LAI	1	20	0.56	0.24	0.08–1.03	—	1	20	0.38	0.23	−0.07 to 0.83	—	—	—	—	—	—	—
*Studies removed in sensitivity analysis*	*Tohen (potential effect of including non‐RC patients)*						*Langosch (RoB/partial remission)*						*Baldessarini (RoB)*					
Mood stabilisers	5	177	0.67	0.14	0.40–0.95	59%^a^	6	192	0.83	0.13	0.57–1.08	51%^a^	—	—	—	—	—	—
Lamotrigine	3	75	0.58	0.21	0.17–0.99	61%^a^	3	75	0.70	0.21	0.29–1.11	59%^a^	—	—	—	—	—	—
Lithium	1	34	0.92	0.21	0.52–1.33	—	2	49	1.01	0.18	0.67–1.36	0%	—	—	—	—	—	—
Divalproex and Na Valproate	1	68	0.77	0.14	0.50–1.00	—	1	68	0.86	0.14	0.58–1.14	—^b^	1	68	0.91	0.14	0.63–1.19	—^c^
*Studies removed in sensitivity analysis*	*Goldsmith 2003 (partial remission)*						*Goldsmith (partial remission) & Langosch (RoB/partial remission)*											
Antidepressants	2	65	0.90	0.19	0.52–1.27	16%^b^	4	101	1.19	0.23	0.75–1.64	52%	—	—	—	—	—	—
Citalopram	1	59	0.99	0.16	0.68–1.30	—	1	59	1.10	0.17	0.78–1.43	—	—	—	—	—	—	—
Escitalopram	1	6	0.48	0.43	−0.36 to 1.33	—	1	6	1.07	0.51	0.06–2.07	—	—	—	—	—	—	—
Paroxetine	—	—	—	—	—	—	1	24	0.90	0.24	0.42–1.37	—	—	—	—	—	—	—
Venlafaxine	—	—	—	—	—	—	1	12	2.48	0.58	1.33–3.62	—	—	—	—	—	—	—
Hormone treatments	—	—	—	—	—	—	2	27	0.60	0.24	0.14–1.06	71%	—	—	—	—	—	—
Levothyroxine	—	—	—	—	—	—	1	15	1.08	0.35	0.39–1.76	—	—	—	—	—	—	—
Triiodothyronine	—	—	—	—	—	—	1	12	0.20	0.32	−0.42 – 0.83	—	—	—	—	—	—	—
Non‐pharmacological	2	16	0.44	0.26	−0.08 to 0.95	0%	—	—	—	—	—	—	—	—	—	—	—	—
CPT	1	7	0.24	0.38	−0.51 to 0.99	—	—	—	—	—	—	—	—	—	—	—	—	—
Bibliotherapy	1	9	0.61	0.36	−0.1 to 1.32	—	—	—	—	—	—	—	—	—	—	—	—	—
Controls^e^	7	347	0.67	0.06	0.55–0.79	0%^a^	11	521	0.60	0.08	0.43–0.76	51%^a^ ^,^ ^f^	4	126	0.33	0.09	0.15–0.51	0%^d^
Placebo							10	496	0.60	0.09	0.42–0.78	55%^a^						
Treatment as usual	—	—	—	—	—	—	1	25	0.55	0.21	0.13–0.97	—	—	—	—	—	—	—
*Studies removed in sensitivity analysis*	*Goldsmith, Tohen 03 (above), Bobo (RoB), Cutler (non‐RC)*						*Goldsmith, Cutler, Muzina 2008 (all mild symptoms)*.						*Baldessarini (above)*					

Abbreviations: CI, confidence interval; CPT, cognitive psychoeducational therapy; ES, effect size; *k*, number of studies; LAI, long‐acting injectable; *N*, number of participants; OFC, olanzapine fluoxetine combination; RC, rapid cycling; RoB, risk of bias.

^a^ Removal of study/ies in sensitivity analysis increased ES (and reduced heterogeneity).

^b^ The only studies assessed for this treatment were flagged as needing checking in sensitivity analyses. The effect on the category‐level analysis was examined and one of two studies (each) were removed for Valproate (depression) and olanzapine (global impression); in both cases, this was because one of the two studies' removal reduced class heterogeneity and one did not; the latter was kept in. For Valproate, removal of the one study increased the ES (due to patients being in partial remission) and for olanzapine removal reduced the ES (due to a potential effect of non‐RC patients being included in the analysis).

^c^ May be over‐estimation of effect (see Supplement 6) but no other studies in category to be compared with.

^d^ Removal of study/ies in sensitivity analysis decreased ES (and reduced heterogeneity).

^e^ Control arms (more studies), were treated slightly differently in sensitivity analyses; initially, all studies which had previously been flagged were removed; these were then re‐added one by one and were kept in where they did not increase I‐squared. If only the same studies (per outcome) had been removed as in the respective active treatment arms, the heterogeneity was clearly higher but effect sizes were equivalent, just with wider CIs. Specifically, the ES for global outcome was 0.64 (73% heterogeneity), for depression was 0.61 (64% heterogeneity) and the mania studies/results were identical to above.

^f^ If excluding two very large outliers (with no a priori reason for exclusion, the results for the control groups would have been *k* = 9, *n* = 370, ES = 0.51, 95% CI 0.38–0.64, i2 = 0%. In this case, the control 95% Cis would not have overlapped with lithium, antidepressant category (and within that, citalopram and venlafaxine) in addition to olanzapine.

#### 4.5.1 Second‐generation antipsychotics

The most commonly studied class of treatments was SGA, with data for all outcomes, and quetiapine being the most common individual treatment.

- Global impression: Quetiapine comprised 4/6 studies and almost 90% of patients on antipsychotics. Both quetiapine/antipsychotics had an overall within‐subjects ES of 0.79 (0% heterogeneity).
- Depression: The depression ES for antipsychotics overall (10 studies) was 0.75, with significant heterogeneity (69%). Quetiapine was studied in 50% of trials, comprising 76% of participants, and its ES was similar to SGAs overall (0.76; 37% heterogeneity). The other antipsychotic trialled in >1 study for the depression outcome was olanzapine with an ES of 1.01 (0% heterogeneity).
- Mania: No treatments were analysed in >1 study with a mania outcome, although the antipsychotic class pooled analysis of 3 studies yielded a higher ES of 1.11 (0% heterogeneity) than for global impression or depression outcomes.

#### 4.5.2 Mood stabilisers

Mood stabilisers was the second‐most commonly studied class of treatment. The only individual treatments analysed in >1 study were lamotrigine and lithium.

- Global impression: The pooled ES was 0.67 across 5 trials (59% heterogeneity). Lamotrigine, examined in three of these trials, had a lower ES (0.58, 61% heterogeneity).
- Depression: The depression ES was higher than for global impression, at 0.83 (51% heterogeneity). Lithium raised the overall ES for this class (1.01; 0% heterogeneity between 2 studies), while lamotrigine lowered it (ES = 0.70, 59% heterogeneity between 3 studies).
- Mania: Only 1 mood stabiliser study (divalproex; ES = 0.91) examined mania outcomes.

#### 4.5.3 Antidepressants

No individual antidepressants were meta‐analysable from >1 study; overall 4 antidepressants were analysed from studies with participants who were depressed at baseline.

1. Global impression: The ES across 2 studies was 0.90 (16% heterogeneity).
2. Depression: The ES across 4 studies was 1.19 (52% heterogeneity).

#### 4.5.4 Other treatment classes (hormone and non‐pharmacological)

Other treatment classes were not assessed for mania; one study (psychological) looked at the global impression and the other (hormonal) focused on depression outcomes.

1. Global impression: One study assessed two psychological interventions (bibliotherapy had a higher ES than cognitive psychoeducational therapy), with a pooled (low) ES of 0.44 (0% heterogeneity).
2. Depression: One trial studied T3 and T4 thyroid medications for depression, with a pooled ES of 0.60 (71% heterogeneity; levothyroxine had a higher ES than triiodothyronine).

#### 4.5.5 Comparator arms

Control interventions had numerically lower ES' than many, but not all, active treatment arms.

1. Global impression: With an ES of 0.67 (0% heterogeneity), the ES was lower than SGAs (pooled, plus quetiapine and olanzapine) and antidepressants (pooled, and citalopram), as well as individual mood stabilisers (lithium, valproate) although the pooled mood stabilisers ES equalled the control ES.
2. Depression: Control arms had a numerically lower ES (0.60, 51% heterogeneity) than mood stabilisers (pooled, plus lithium, lamotrigine and valproate medications), antipsychotics (pooled, plus quetiapine and olanzapine) and antidepressants (pooled, citalopram, escitalopram, paroxetine, and venlafaxine). The control arms ES was equal to thyroid treatments (0.60).
3. Mania: The placebo ES was lower for mania (0.33, 0% heterogeneity) than for other outcomes, and when compared to the only other class of treatments assessed (antipsychotics pooled, plus quetiapine, olanzapine/OFC, and aripiprazole).

Although control ES' were not substantially lower than many active treatments, due to the higher number of studies (alongside removal of more studies in sensitivity analyses), the confidence intervals were narrower. When comparing overlap of confidence intervals between active and control arms, no treatments appeared significantly different from controls in global impression outcomes, but for depression olanzapine (3 studies), citalopram (1 study) and venlafaxine (1 study) appeared significantly more efficacious than controls. For mania, all active treatments analysed were significantly more effective than controls (quetiapine, olanzapine, aripiprazole).

### 4.6 Studies not eligible for meta‐analysis

Of the 30 studies, 23 were included in meta‐analyses. Four were not analysed because they did not report primary outcomes (continuous efficacy data) required for analysis; their findings are summarised below. The other three were not meta‐analysed because they only reported other outcomes for which insufficient data were available to be analysed; these outcomes that were not meta‐analysed are presented in Supplement 7 (for these three studies along with another five studies that had also been included in meta‐analyses).

Amsterdam et al. (2017) compared 12 weeks of venlafaxine to lithium for depressed participants, finding that compared to lithium, venlafaxine had both a higher response rate (59% vs. 39%) but also higher discontinuation rate (59% vs. 22%).^39^

Amsterdam et al. (2013) compared up to 1 year's treatment of fluoxetine, lithium and placebo for euthymic participants. Relapse rates were approximately equivalent across each arm (29–35%).^34^

Post et al. (2006) compared bupropion, venlafaxine and sertraline adjunct to mood stabilisers over 10 weeks for depressed participants. The proportion of participants relapse/switching was low for sertraline (8%) and bupropion (14%), and higher for venlafaxine (43%). Whereas the three medications were similar in this sense for participants without RCBD, the risk of manic switch was lower with bupropion than venlafaxine for those with RCBD.^40^

Keck et al. trialled an agent of a different class ‐ ethyl‐eicosapentanoate (EPA) compared with placebo for 16 weeks, finding no efficacy differences between groups (finding a higher manic switch rate [25%] with EPA than placebo [19%]).^41^

### 4.7 Secondary outcomes

#### 4.7.1 Binary efficacy outcome variables

As can be observed in Supplement 5, the proportion of participants responding/remitting or relapsing varied widely. Although these rates are difficult to interpret without considering treatment duration, baseline characteristics and other factors, the highest response rate of all arms reported was 77%; 77% responded to 8 weeks of low‐dose quetiapine monotherapy for BD‐1 patients with depression (vs. 57% placebo response)^42^ and 77% also responded to 3–4 weeks of olanzapine for acute mania (or mixed states), compared to 50% placebo (both augmenting usual care). However, it is also notable that 57% of RCBD participants in the quetiapine study experienced a serious adverse event.^43^ The highest remission rate (77%) was identified for already partially‐remitted participants after 12 weeks of lamotrigine (vs. 66% on placebo), although only 41% of lamotrigine remission was sustained over the total of 26 weeks (vs. 26% sustained remission on placebo).^44^ Furthermore, it is relevant that another lamotrigine study for people with depression reported only a 13% remission after 12 weeks.^45^ The lowest relapse rate in active treatment arms was 14% in participants taking long‐term maintenance aripiprazole after euthymia (compared with 43% in the placebo arm).^27^

#### 4.7.2 Tolerability and acceptability

These data are summarised in Table 2. Tolerability and acceptability were defined differently between studies and were not sufficiently homogeneous to be considered in quantitative meta‐analyses. Importantly, tolerability and acceptability data specifically pertaining to subjects with RC were limited. Across all studies, the highest discontinuation rates were from two long‐term studies: first, 1 year of lithium (89%) and placebo (88%, vs. fluoxetine, 50%) although this included only 25 patients (8 fluoxetine, 9 lithium and 8 placebo)^34^ and secondly over 47 weeks of olanzapine (85%) and divalproex (84%)^46^ though the latter rate included both patients with and without RCBD.

The highest adverse event (AE) rate in specifically RCBD participants was reported in a 52 week study of patients on quetiapine (86%).^29^ Of studies specifically reporting the proportion of participants with any AE, the lowest rate for active treatments was in aripiprazole maintenance therapy (7%).^27^

## 5 DISCUSSION

A likely reason for the absence of a previous quantitative meta‐analyses collating this literature is the scant evidence base, with traditional (between subjects) meta‐analyses requiring a common comparator in each treatment trial. We sought to overcome this by employing within‐subjects meta‐analyses for each intervention with available data, therefore not excluding studies that have compared a range of interventions with one another or uncommon comparators. This approach has, in our view, enabled a meta‐analysis where one would otherwise be considered not viable. This is especially relevant for limited evidence bases where clinical implications of relative treatment differences could impact routine care.^17^, ^23^

### 5.1 Summary of main findings

In this first meta‐analysis of treatments for RCBD we identified 30 studies. The only class with evidence of effectiveness over placebo for treating mania (in more than one trial per class) was SGAs, particularly quetiapine, olanzapine and aripiprazole. Quetiapine was the most widely studied antipsychotic and demonstrated consistently large ES's across outcomes. This evidence of quetiapine efficacy to treat both affective poles aligns with evidence in non‐rapid cycling BD.^47^ Olanzapine also showed a particularly large effect sizes though we note possible allegiance bias across SGAs. Lastly, it should be taken into consideration that aripiprazole results were obtained from fewer participants. Mood stabilisers and antidepressants had a numerically higher ES than SGAs for depression, but none of these classes appeared to statistically outperform placebo (although see below considerations related to the validity of this approach to determining statistical significance). The same was true for global impression outcomes, where numerically mood stabilisers were both lower than SGAs and antidepressants. It must be emphasised that the evidence for patients who were manic at baseline is limited (unsurprisingly, entirely so for antidepressants, and largely also for mood stabilisers). Manic switch rates could not be assessed due to the lack of reported data but these appeared relatively low for SSRI's (citalopram, escitalopram, sertraline, fluoxetine) and bupropion, although markedly higher for venlafaxine. Psychological and hormone therapies have only been trialled in a very small number of participants, with uncertain efficacy, and no non‐psychological non‐pharmacological therapies were trialled (despite previous evidence indicating promise for neuromodulation, that is, vagus nerve stimulation^48^).

### 5.2 Antidepressants in RCBD

There is a common view that antidepressants can worsen rapid cycling in people with BD, exemplified by STEP‐BD findings, which examined outcomes over 3 years and found that continuing (vs. discontinuing) antidepressants was associated with stark increases in the number of depressive episodes.^49^, ^50^ In one sense, our finding may refute the assumption that antidepressants cannot be beneficial in people with RCBD. The antidepressants included here were citalopram, escitalopram, fluoxetine, paroxetine, sertraline and bupropion (1 study each) and venlafaxine in 3 studies. It is worth considering both the type of antidepressant, and the duration of treatment here. However, we did not analyse manic switch rates, or the number of episodes in a long follow‐up period; the benefits we report here are from single outcomes in (mostly) short term studies. We note three key considerations here: 1) patient factors; some may benefit and there is a need to contemplate for example predominant polarity and episode density in thinking about antidepressant suitability; 2) outcome factors; assessing global response in trials, and the feasibility of close monitoring in real world care; 3) duration factors; trials need to understand the effects of long‐term antidepressant use especially in RCBD patients. These are particular concerns with regard to antidepressants, although they are factors common to other treatment modalities (see below; “rethinking the evidence of RCBD therapeutics”).

SSRIs appear moderately effective and moderately safe, even in the longer term: the included SSRI trials randomised small samples, but none of the five reported particularly high manic switch rates (with escitalopram,^28^ citalopram,^49^ fluoxetine,^34^ paroxetine^35^ or sertraline^40^) relative to other arms, albeit over varying time periods (up to 1 year). In the latter study, sertraline had higher discontinuation rates due to adverse events than bupropion or venlafaxine, but lower relapse rates, while bupropion had the lowest discontinuation rate overall, and venlafaxine had the highest relapse and overall discontinuation rate.^40^ The idea of bupropion as a relatively safe and effective antidepressant has also previously showed promise for reduced cycling compared to other antidepressants and as such warrants further investigation.^51^ Conversely, and aligning with the study mentioned above,^40^ the other two venlafaxine studies reported high depression response rates but also high manic switch and discontinuation with this medication.^33^, ^39^, ^40^

A determinant of stability in BD related to antidepressants is the specific medication *combination* taken, where an antidepressant paired with an antimanic agent can promote extended periods of recovery^52^; a factor we were unable to investigate in this review, particularly given the limited evidence base.

### 5.3 Mood stabilisers and the lack of antimanic evidence

As above, the paucity of studies examining antimanic effects of mood stabilisers in RCBD is concerning and contrasts the evidence base for BD in general, where lithium and valproate – at least – have a high evidence grade in guidelines^12^ and clear efficacy for mania in meta‐analyses.^53^ Our mania meta‐analysis contained only one trial assessing divalproex and none for lithium or lamotrigine (one of the included lithium studies did examine mania with insufficient results reported to meta‐analyse, and with only 7 patients studied.)

One lithium (34 patients) and one valproate (68 patients) study were meta‐analysable for global impression outcome, and even for depression, only two lithium studies (49 patients) and one valproate (68 patients; a second valproate study was excluded due to high RoB) was analysed. There was less uncertainty for lamotrigine, with three trials pooled, although this still comprised only 75 participants in total.

### 5.4 Rapid cycling versus non‐rapid cycling; differences in treatment response

A poorer response in RCBD compared with non‐rapid cycling patients would not be unexpected, given the nature of this illness and previous evidence both for individual treatments, for example, Ref. 54, and across the range of BD medications.^8^ Of our included studies including both RC and non‐RC participants (irrespective of treatments), 4 reported a worse response in rapid cyclers, 9 identified non‐significant differences between RC and non‐RC participants, and 1 study found a better response in RC patients (to lithium and venlafaxine). The treatments/outcomes in the studies that reported a poorer outcome in RCBD versus non‐rapid cycling subgroups were: 1) in mania following olanzapine and divalproex,^46^ 2) manic symptoms after citalopram,^55^ 3) depression after both olanzapine (with and without fluoxetine) and placebo^25^ and 4) mania after both quetiapine and placebo.^26^ Another study found a significant benefit of quetiapine over placebo for non‐RC but not RC participants, although it is not clear whether this indicates a reduced response to quetiapine or increased placebo response in participants with RCBD.^35^ The other notable finding here was from a study of bupropion and venlafaxine, where depressed individuals with RCBD responded better to bupropion than venlafaxine but no between‐treatment effect was evident for non‐RC participants.^40^ Regarding the latter, we have already mentioned the evidence for high manic switch associated with venlafaxine compared with lower switch rates with bupropion; it may be that, since RCBD patients are vulnerable to episode shifts, that these effects are magnified in this population.

There may be a duration effect, where people with RCBD respond more poorly to longer‐term but not necessarily shorter‐term treatment, and we were not able to obtain sufficient evidence to establish this, although our findings included one report of some olanzapine outcomes being better in the short‐term and poorer in the long‐term for people with RCBD compared with non‐rapid cycling BD.^30^, ^43^, ^56^, ^57^ An overall duration effect was indicated, with the longer studies having the smallest effect sizes, followed by the shorter studies, but as above this is indicative only and requires a greater evidence base to examine in detail. We acknowledge that the separation of short, medium and longer‐term studies (<6 weeks, 6–51 weeks, 52+ weeks) uses somewhat arbitrary durations, particularly the wide range for studies that were not specifically short or long term.

### 5.5 Strengths and limitations

This is the largest synthesis of treatment efficacy for people with RCBD. A strength is the broad inclusion criteria: we included non‐pharmacological as well as pharmacological therapies, and we considered studies including participants with/without RC where feasible (i.e., if RCBD participants formed the majority of the sample and/or if there was an efficacy indication specifically for RCBD).

A key drawback of inclusivity is increased heterogeneity. This was overcome to some extent by our in‐depth consideration of potential subgroups and sources of heterogeneity. After removing outlying studies (for example where patients were not presenting with significant symptoms at baseline) we observed reduced heterogeneity which was not significant in 10 out of 12 class‐level analyses conducted. This approach to sensitivity meta‐analyses has been deemed appropriate where studies are methodologically variable.^58^ One of the sources of heterogeneity investigated was where some participants without rapid cycling had been included in original analyses, and this only altered meta‐analysis heterogeneity in one out of five studies tested. It may be argued that including some individuals without rapid cycling, even despite this, is a weakness of our analysis but it was decided in order to maximise the data that could be included in cases where it did not influence our effect sizes. This was due to our understanding of the bias risk introduced in meta‐analysis by not including all relevant available data.^59^

In other respects, the review was less inclusive; the decision to include only randomised controlled trials was made to minimise bias and methodological heterogeneity and specifically not to over‐inflate effect sizes.^60^ Another factor which has been reported to inflate effect sizes, and one that is not typically included in risk of bias assessments, is the potential for allegiance biases (e.g., funding or sponsoring of studies by pharmaceutical manufacturers).^61^ Approximately half of our included studies were indicated to have a potential allegiance bias and this differed between intervention category; antidepressant and mood stabiliser specific studies had a potential allegiance bias in one third of studies, while this rate was 57% of studies examining multiple classes (which might magnify between‐treatment effects) and 100% of antipsychotic studies. Though we are unable to infer any influence this may have had in meta‐analyses, this further limits the evidence base, at least for mania, where SGAs were the only well studied class of treatments.

Notably, most included studies were not specifically trialling treatment for RCBD and many data are procured from secondary analyses.^62^, ^63^, ^64^, ^65^, ^66^, ^67^ Although we undertook handsearching to maximise the evidence retrieved, we were unable to formally assess the possibility of publication bias which may overinflate a true effect size. Other limits to the validity of our findings include an under‐representation of some areas of the globe, with most evidence gathered in North America, and even within‐samples are unlikely to be truly representative of local demographics.

Where studies reported outcomes at several different time points, we included outcomes at the last post‐treatment time point. This approach was chosen to prioritise long‐term outcomes, particularly important given the clinical course of RCBD. However, this may have resulted in shorter‐term rapid effects of medications being under‐represented.

We considered a modified intention to treat (ITT) approach to analyses as equivalent to a full ITT analysis where few participants were excluded from analyses, usually only where it is not possible to infer any information regarding their state after baseline. >5% of participants excluded, despite claims of ITT/mITT resulted in a slightly higher risk of bias rating, and use of a completer analysis was coded as substantively high risk of bias. However, our pre‐selection of 5% as a threshold rate of exclusion could be argued as arbitrary.

Methodologically, we emphasise that the current methods employed within‐ rather than between‐subjects meta‐analyses; thus, our analyses do not permit direct comparisons between groups, and our effect sizes incorporate a variety of time‐ related factors such as natural recovery. This has been legitimately criticised,^68^ but with a small evidence base such as this, employing pre‐post meta‐analysis not only permitted a greater inclusion of available data (for example, studies without a common comparator) and allowed indications of a higher number of interventions' effects. Furthermore, pre‐post effect sizes have the additional benefit of delivering good clinical face validity as an estimate of the magnitude of effects observed with treatment in practice, including both those specific to the individual modality as well as the non‐specific effects.^17^, ^23^ This can be particularly applicable to patient groups who have decreased rates of recovery during the natural course of illness, such as rapid cycling.

Finally, we were not able to account for the wide range of continuation/adjunctive therapies participants were taking and specific combinations may be particularly beneficial or problematic. This is something that requires further examination.

### 5.6 Rethinking the evidence for RCBD therapeutics

When considering treatment for a patient with rapid cycling, a clinician's priority is ensuring an enduring response; a clear goal is for them to no longer meet criteria for the rapid cycling specifier, and further to prevent future RCBD relapse. By definition, treatment follow‐up of at least 1 year would be needed to ascertain true RCBD remission. This is challenging for developing an evidence base, with long‐term trials being costly to undertake and suffering from issues with participant attrition and subsequent bias. This is a key reason for the paucity of trials, with most evidence derived from retrospective cohort studies, but long‐term trials are critical.^69^ The drawbacks of the current RCTs, therefore, being few with inadequate follow‐up durations, limit clinically meaningful conclusions that can be drawn from our findings. In this sense, therefore, innovation is needed in clinical trial designs to increase the rigour and consequent quality of the evidence base. Another consideration that clinicians make in selecting treatments is the outcome, and we emphasise the need for future trials assessing patient centred outcomes that incorporate psychosocial functioning and quality of life in addition to reduction of episode burden (episode number and severity, and switches).

### 5.7 Future avenues and implications

Clearly, elucidating optimal pharmacological and non‐pharmacological treatment strategies for people with RCBD is a clinical priority that remains. Further research is required across the board. As well as expanding research on the gold standard treatment options for people with BD overall (particularly, for example lithium and anticonvulsants for mania), hormone and psychological treatments are virtually unstudied for this population. This is despite evidence that people with RCBD frequently present with thyroid dysfunction^70^ (although at least one review has refuted an association between hypothyroidism and RC^71^) and with adjunct levothyroxine showing much promise in non‐randomised studies.^72^ Specific non‐pharmacological therapies trialled for RCBD should include psychoeducation)^73^ and electro‐convulsive therapy,^74^ which can have enduring prophylactic effects in BD. Newer antipsychotics (e.g., cariprazine) and a wider range of antidepressants (e.g., vortioxetine) also warrant investigation. It is emphasised that trials recruiting an adequate sample size of RCBD patients are required across the board. For both pharmacological and non‐pharmacological interventions, future research also needs to focus on the tolerability and acceptability of treatments; data availability specifically for participants with RCBD was low in the studies included in our review.

In the introduction section of our review, we stated that treatment guidelines do not contain specific recommendations for people with RCBD. This clearly needs to change, and although we are in dire need of expanding this evidence base, our findings may be considered in updating BD management guidelines. Unlike national/international guidelines, the Maudsley Prescribing Guidelines (London)^75^ do currently have a section focused on RCBD where they recommend that: 1) All patients are first withdrawn from antidepressants (this is not necessarily supported in our review); 2) Mood stabiliser combinations should then be considered (we support promise for lithium and valproate medications); 3) Augmentation of mood stabilisers should be considered with preferred agents listed as quetiapine, olanzapine and aripiprazole (our findings support this overall, although aripiprazole evidence for depression is lacking). They also advise considering other adjunct therapies including lamotrigine (which we found to have been more investigated than other options), risperidone or thyroxine (which we have found limited evidence to support), clozapine, levetiracetam, nimodipine, or topiramate (which we identified no RCT evidence of.)

### 5.8 Concluding remarks

Ideally here we would highlight the best interventions for treating RCBD. “Good” options not only require low heterogeneity and high effect sizes but also a high number of patients studied in long‐term, low risk of bias trials, and with evidence of acceptable tolerability. No treatments/outcomes met all of these criteria. Discounting risk of bias from the above criteria, quetiapine and olanzapine appear to be good options, but risk of bias is clearly critical here and olanzapine in particular has been downgraded in other guidelines due to lack of evidence for depression and tolerability concerns.^7^ Instead relaxing the criterion of data quantity, lithium and valproate appear preferable. Otherwise, we, like previous syntheses, do not find a definitive benefit of one treatment over another for RCBD, which is likely in part due to the amount of available evidence alongside heterogeneity of study designs, specific intervention features (e.g., dose, duration) and patients.^8^, ^9^

## AUTHOR CONTRIBUTIONS

The following authors contributed to the following roles in this research: Rebecca Strawbridge and Allan H. Young in formulating the research question and designing the study; Rebecca Strawbridge, Suman Kurana, Jess Kerr‐Gaffney, Kenneth R Kaufman in review conduct, Rebecca Strawbridge in data analysis, Rebecca Strawbridge, Nefize Yalin and Sameer Jauhar in interpreting analyses, Suman Kurana and Rebecca Strawbridge in the article's first draft, all authors in critically revising and finally approving the manuscript.

## FUNDING INFORMATION

This work is supported by the National Institute for Health Research (NIHR) Maudsley Biomedical Research Centre at South London and Maudsley NHS Foundation Trust and King's College London. The views expressed are those of the authors and not necessarily those of the NIHR or the Department of Health and Social Care.

## CONFLICT OF INTEREST

In the last 3 years: Rebecca Strawbridge declares an honorarium from Lundbeck; Allan H. Young declares honoraria for speaking from Astra Zeneca, Lundbeck, Eli Lilly, Sunovion, honoraria for consulting from Allergan, Livanova and Lundbeck, Sunovion, Janssen, and research grant support from Janssen; Sameer Jauhar has received honoraria for educational talks given for Lundbeck, Sunovian and Janssen, on antipsychotics; Nefize Yalin has worked on studies conducted together with Janssen Cliag, Corcept Therapeutics and COMPASS Pathways. No other conflicts of interest are declared.

## Supporting information


**Appendix S1** Supplementary Information.

## Data Availability

The data that support the findings of this study are available from the corresponding author, Rebecca Strawbridge, upon reasonable request and also available in the supplementary material of this article.
