# Supplementary material for: A systematic review and meta‐analysis of treatments for rapid cycling bipolar disorder
Source: Acta Psychiatr Scand. 2022 Jul 20;146(4):290–311. doi: 10.1111/acps.13471 (PMC9796364; doi:10.1111/acps.13471)
Supplement: Supplementary file 1 — Appendix S1 Supplementary Information. [file ACPS-146-290-s001.docx]

**Supplementary material**

**Supplement 1:** Secondary outcome data extraction and presentation

Secondary outcome 1: binary efficacy outcomes

Most often this was reported as % of the sample responding to each treatment (usually defined as reducing symptom severity scores by >50% during treatment, but presented here according to definitions used in original articles). If this was not available, the % of participants meeting criteria for symptom remission after treatment was extracted and presented. If this was not available, a relapse % (particularly for populations euthymic at baseline) was extracted and presented.

The results for this outcome are presented in Supplement 5.

Secondary outcome 2: tolerability outcomes

A measure of tolerability was recorded where available. Tolerability outcomes were selected in a hierarchical fashion. Where available, the mean (and SD or equivalent) number of adverse events per participant was prioritised. If this was not available the % of participants with an adverse event was prioritised, followed by the % of participants discontinuing treatment due to tolerability/adverse events, the % of participants with a serious adverse event, the mean (and SD or equivalent) number of serious adverse events per participant, the general validated side effect scale score and SD or equivalent and the % reporting specific symptoms as side effects. Where separate results were not reported for subjects with RC, data regarding each treatment group was extracted and presented.

The results for this outcome are presented in Table 2.

Secondary outcome 3: acceptability outcomes

A measure of acceptability was recorded where available. Acceptability outcomes were selected in a hierarchical fashion. Where available, the % of participants discontinuing treatment for any reason (per treatment groups) was prioritised, followed by the % of participants discontinuing study for any reason (per treatment group), if not as before but whole sample (regardless of treatment group). Where separate results were not reported for subjects with RC, data regarding each treatment group was extracted and presented.

The results for this outcome are presented in Table 2.

**Supplement 2:** Delineation of articles and studies examined in this review (where other than one article per one study)

Greyed out papers were not themselves eligible for inclusion in the current review

| Secondary analyses included in this review | Original randomised controlled trial |
| --- | --- |
| Goldberg, J. F., Bowden, C. L., Calabrese, J. R., Ketter, T. A., Dann, R. S., Frye, M. A., ... & Post, R. M. (2008). Six-month prospective life charting of mood symptoms with lamotrigine monotherapy versus placebo in rapid cycling bipolar disorder. *Biological psychiatry*, *63*(1), 125-130. | Calabrese, J. R., Suppes, T., Bowden, C. L., Sachs, G. S., Swann, A. C., McElroy, S. L., ... & Monaghan, E. T. (2000). A double-blind, placebo-controlled, prophylaxis study of lamotrigine in rapid-cycling bipolar disorder. *Journal of Clinical Psychiatry*, *61*(11), 841-850. |
| Vieta, E., Calabrese, J. R., Hennen, J., Colom, F., Martínez-Arán, A., Sánchez-Moreno, J., ... & Baldessarini, R. J. (2004). Comparison of rapid-cycling and non-rapid-cycling bipolar I manic patients during treatment with olanzapine: analysis of pooled data. *Journal of Clinical Psychiatry*, *65*(10), 1420-1428.  Baldessarini, R. J., Hennen, J., Wilson, M., Calabrese, J., Chengappa, R., Keck Jr, P. E., ... & Tohen, M. (2003). Olanzapine versus placebo in acute mania: treatment responses in subgroups. *Journal of clinical psychopharmacology*, *23*(4), 370-376.  Shi, L., Schuh, L. M., Trzepacz, P. T., Huang, L. X., Namjoshi, M. A., & Tohen, M. (2004). Improvement of Positive and Negative Syndrome Scale cognitive score associated with olanzapine treatment of acute mania. *Current medical research and opinion*, *20*(9), 1371-1376. | Tohen, M., Sanger, T. M., McElroy, S. L., Tollefson, G. D., Chengappa, K. R., Daniel, D. G., ... & Olanzapine HGEH Study Group. (1999). Olanzapine versus placebo in the treatment of acute mania. *American Journal of Psychiatry*, *156*(5), 702-709.  **AND**  Tohen, M., Jacobs, T. G., Grundy, S. L., McElroy, S. L., Banov, M. C., Janicak, P. G., ... & Olanzapine HGGW Study Group. (2000). Efficacy of olanzapine in acute bipolar mania: a double-blind, placebo-controlled study. *Archives of general psychiatry*, *57*(9), 841-849. |
| Sanger, T. M., Tohen, M., Vieta, E., Dunner, D. L., Bowden, C. L., Calabrese, J. R., ... & Breier, A. (2003). Olanzapine in the acute treatment of bipolar I disorder with a history of rapid cycling. *Journal of affective disorders*, *73*(1-2), 155-161. | **ONLY (from above)**  Tohen, M., Sanger, T. M., McElroy, S. L., Tollefson, G. D., Chengappa, K. R., Daniel, D. G., ... & Olanzapine HGEH Study Group. (1999). Olanzapine versus placebo in the treatment of acute mania. *American Journal of Psychiatry*, *156*(5), 702-709. |
| Suppes, T., Brown, E., Schuh, L. M., Baker, R. W., & Tohen, M. (2005). Rapid versus non-rapid cycling as a predictor of response to olanzapine and divalproex sodium for bipolar mania and maintenance of remission: post hoc analyses of 47-week data. *Journal of affective disorders*, *89*(1-3), 69-77. | Tohen, M., Ketter, T. A., Zarate, C. A., Suppes, T., Frye, M., Altshuler, L., ... & Baker, R. W. (2003). Olanzapine versus divalproex sodium for the treatment of acute mania and maintenance of remission: a 47-week study. *American Journal of Psychiatry*, *160*(7), 1263-1271. |
| Vieta, E., Calabrese, J. R., Goikolea, J. M., Raines, S., Macfadden, W., & BOLDER Study Group. (2007). Quetiapine monotherapy in the treatment of patients with bipolar I or II depression and a rapid‐cycling disease course: a randomized, double‐blind, placebo‐controlled study. *Bipolar disorders*, *9*(4), 413-425. | Calabrese, J. R., Keck Jr, P. E., Macfadden, W., Minkwitz, M., Ketter, T. A., Weisler, R. H., ... & BOLDER Study Group. (2005). A randomized, double-blind, placebo-controlled trial of quetiapine in the treatment of bipolar I or II depression. *American Journal of Psychiatry*, *162*(7), 1351-1360. |
| Lorenzo‐Luaces, L., Amsterdam, J. D., Soeller, I., & DeRubeis, R. J. (2016). Rapid versus non‐rapid cycling bipolar II depression: response to venlafaxine and lithium and hypomanic risk. *Acta Psychiatrica Scandinavica*, *133*(6), 459-469. | Amsterdam, J. D., Lorenzo‐Luaces, L., & DeRubeis, R. J. (2017). Comparison of treatment outcome using two definitions of rapid cycling in subjects with bipolar II disorder. *Bipolar disorders*, *19*(1), 6-12. |
| Muzina, D. J., Momah, C., Eudicone, J. M., Pikalov, A., McQuade, R. D., Marcus, R. N., ... & Carlson, B. X. (2008). Aripiprazole monotherapy in patients with rapid‐cycling bipolar I disorder: an analysis from a long‐term, double‐blind, placebo‐controlled study. *International journal of clinical practice*, *62*(5), 679-687. | Keck Jr, P. E., Calabrese, J. R., McQuade, R. D., Carson, W. H., Carlson, B. X., Rollin, L. M., ... & Sanchez, R. (2006). A randomized, double-blind, placebo-controlled 26-week trial of aripiprazole in recently manic patients with bipolar I disorder. *Journal of Clinical Psychiatry*, *67*(4), 626-637. |
| Suppes, T., Eudicone, J., McQuade, R., Pikalov III, A., & Carlson, B. (2008b). Efficacy and safety of aripiprazole in subpopulations with acute manic or mixed episodes of bipolar I disorder. *Journal of affective disorders*, *107*(1-3), 145-154 | Sachs, G., Sanchez, R., Marcus, R., Stock, E., McQuade, R., Carson, W., ... & Iwamoto, T. (2006). Aripiprazole in the treatment of acute manic or mixed episodes in patients with bipolar I disorder: a 3-week placebo-controlled study. *Journal of Psychopharmacology*, *20*(4), 536-546.  Keck Jr, P. E., Marcus, R., Tourkodimitris, S., Ali, M., Liebeskind, A., Saha, A., ... & Aripiprazole Study Group. (2003). A placebo-controlled, double-blind study of the efficacy and safety of aripiprazole in patients with acute bipolar mania. *American Journal of Psychiatry*, *160*(9), 1651-1658. |
| Post, R. M., Altshuler, L. L., Leverich, G. S., Frye, M. A., Nolen, W. A., Kupka, R., ... & Mintz, J. (2006). Mood switch in bipolar depression: comparison of adjunctive venlafaxine, bupropion and sertraline. *The British Journal of Psychiatry*, *189*(2), 124-131. | Post, R. M., Altshuler, L. L., Frye, M. A., Suppes, T., Rush, A. J., Keck Jr, P. E., ... & Nolen, W. A. (2001). Rate of switch in bipolar patients prospectively treated with second‐generation antidepressants as augmentation to mood stabilizers. *Bipolar disorders*, *3*(5), 259-265. |

**Supplement 3:** Additional characteristics of studies

| Reference | Intervention | Continent | Continuation Tx | Comorbidity |  | Mean age | % Female |
| --- | --- | --- | --- | --- | --- | --- | --- |
| Kemp et al. 2012 | Lamotrigine v Placebo | NAm | Divalproex, Lithium | Some |  | 36 / 43 | 52% / 58% |
| Wang et al. 2010 | Lamotrigine v Placebo | NAm | Divalproex, Lithium | Prominent |  | 35 / 38 | 39% / 39% |
| Suppes et al. 2008 | Lamotrigine v Lithium | NAm | NA | None Prominent |  | 37* / 36* | 68%* / 57%* |
| Calabrese et al. 2000  ^a^ | Lamotrigine v Placebo | NAm | NA | None prominent |  | 39 / 38 | 54% / 59% |
| Goldsmith et al. 2003 | Lamotrigine v Placebo | Aus, Eur, NAm | TAU | None prominent |  | 43 / 44 | 56% / 58% |
| Walden et al. 2000 | Lithium v Lamotrigine | NR | NA | None prominent |  | 39 / 42 | 57% / 71% |
| Amsterdam et al. 2017 ^a^ | Venlafaxine v Lithium | NAm | NA | None Prominent |  | 40 | 61% |
| Amsterdam et al. 2013 | Fluoxetine v Lithium v Placebo | NAm | NA | None prominent |  | 35^d^ | 36%^d^ |
| Amsterdam et al. 2009 | Venlafaxine v Lithium | NAm | NA | Some |  | 31^d^ | 70%^d^ |
| Tohen et al. 2006 | Olanzapine v Placebo | NAm, Eur | NA | None Prominent |  | 41* / 40* | 61%* / 61%* |
| Suppes et al. 2005^a^ | Olanzapine v Divalproex | NAm | NA | None Prominent |  | 39 / 39 | 57 / 56 |
| Baldessarini et al. 2003 ^a^ | Olanzapine v Placebo | NAm | NA | None Prominent |  | 39* | 42%^d^ |
| Sanger et al. 2003 ^a^ | Olanzapine v Placebo | NAm | NA | None prominent |  | 39 / 37 | 47% / 42% |
| Tohen et al. 2003 | Olanzapine v Placebo v OFC | 13 countries | TAU | None Prominent |  | 42* / 42* / 40* | 62%* / 63%* / 67%* |
| Suppes et al. 2014 | Quetiapine v Placebo | NAm | NA | None Prominent |  | 39* / 40* | 66%* / 63%* |
| Cutler et al. 2011 | Quetiapine v Placebo | NAm | NA | None Prominent |  | 41* / 41* | 38%* / 42%* |
| Langosch et al. 2008 | Quetiapine v Na Val | Eur | NA | None prominent |  | 45 / 38 | 71% / 44% |
| Vieta et al. 2007^a^ | Quetiapine v Placebo | NAm | NA | None Prominent |  | 35/34 / 37 | 52/61% / 74% |
| Thase et al. 2006 | Quetiapine v Placebo | NAm | NA | None Prominent |  | 37/38* / 38* | 56/55%* / 60%* |
| Muzina et al. 2008^a^ | Aripiprazole v Placebo | NAm, SAm | NA | None Prominent |  | 38 / 39 | 64% / 71% |
| Suppes et al. 2008b^a^ | Aripiprazole v Placebo | NAm | NA | None Prominent |  | 39* / 40* | 53%* / 55%* |
| Bobo et al. 2011 | Risperidone LAI v TAU | NAm | TAU | Prominent |  | 43 / 38 | 70% / 64% |
| Ghaemi et al. 2021 | Citalopram v Placebo | NAm | TAU | None Prominent |  | 41* / 42* | 63%* / 54%* |
| Parker et al. 2006 | Escitalopram v Placebo | Eur, Aus | NA | None-prominent |  | 29 | 50%^d^ |
| Post et al. 2006^a^ | Venlafaxine v Bupropion v Sertraline | Nam, Eur | TAU | None prominent |  | 41* / 43* / 41* | 55%* / 45%* / 51%* |
| Walshaw et al. 2018 | Levothyroxine v T3 v Placebo | NAm | Lithium | None prominent |  | 36 / 37 / 35 | 62% / 60% / 89% |
| Keck et al. 2006 | Ethyl-EPA v Placebo | NAm | TAU | None prominent |  | 42 / 44 | 42% / 61% |
| Lenz et al. 2016 ^b^ | CPT v Bibliotherapy | Eur | NA | Some |  | 39* / 42* | 60%* / 58%* |

**^a^** Secondary analyses/multiple articles per study (see Supplement 2). ^b^ ICD rather than DSM criteria.

Abbreviations: OFC = olanzapine fluoxetine combination; LAI = long-acting injectable; TAU = treatment as usual; T_3_ = triiodothyronine; ethyl-EPA = ethyl-eicosapentanoate; CPT = cognitive psychoeducational therapy; NAm = North America, Aus = Australasia, Eur = Europe; NR = not reported, SAm = South America, NA = not applicable.

**Supplement 4:** Risk of bias assessment

| Study | Potential sources of bias considered | | | | | | | | | Overall RoB summary |
| --- | --- | --- | --- | --- | --- | --- | --- | --- | --- | --- |
|  | **1** | **2** | **3** | **4** | **5** | **6** | **7** | **8** | **9** |  |
| Lamotrigine |  |  |  |  |  |  |  |  |  |  |
| Kemp et al., 2012 | ? | ? | + | + | + | + | + | ? | + | Low |
| Wang et al., 2010 | ? | ? | + | + | + | + | + | ? | + | Low |
| Suppes et al., 2008 | ? | ? | + | ? | - | + | + | ? | + | Low |
| Calabrese et al., 2000 ^a^ | ? | ? | - | + | + | + | + | ? | - | Moderate |
| Goldsmith et al., 2003 | ? | ? | - | + | + | + | + | ? | - | Moderate |
| Walden et al., 2000 | - | ? | - | - | + | + | ? | ? | + | Moderate |
| Lithium |  |  |  |  |  |  |  |  |  |  |
| Amsterdam et al., 2017 ^a^ | ? | ? | - | + | + | + | + | + | + | Low |
| Amsterdam et al., 2013 | ? | ? | + | + | + | + | + | - | + | Low |
| Amsterdam et al., 2009 | ? | ? | - | - | + | + | ? | ? | + | Moderate |
| Olanzapine |  |  |  |  |  |  |  |  |  |  |
| Tohen et al., 2006 | + | ? | - | + | + | + | + | - | - | Moderate |
| Suppes et al., 2005^a^ | ? | ? | - | + | + | + | + | ? | - | Moderate |
| Baldessarini et al. 2003 / Sanger et al., 2003 ^a^  ^[NB two trials pooled with same design/RoB]^ | - | - | - | + | - | + | + | ? | - | High |
| Tohen et al., 2003 | - | - | + | + | + | + | + | ? | - | Moderate |
| Quetiapine |  |  |  |  |  |  |  |  |  |  |
| Suppes et al., 2014 | ? | ? | - | + | + | + | + | + | - | Moderate |
| Cutler et al., 2011 | + | + | - | + | + | + | + | - | - | Moderate |
| Langosch et al., 2008 | ? | ? | + | - | + | - | - | + | - | High |
| McElroy et al. 2010 | ? | + | + | + | + | + | + | ? | - | Low |
| Vieta et al. 2007^a^ | + | + | - | + | + | ? | + | ? | - | Moderate |
| Thase et al., 2006 | + | + | + | + | + | + | + | + | - | Low |
| Aripiprazole |  |  |  |  |  |  |  |  |  |  |
| Muzina et al., 2008^a^ | ? | ? | - | + | + | + | + | ? | - | Moderate |
| Suppes et al., 2008b^a^  ^[NB two trials pooled with same design/RoB]^ | ? | ? | - | + | - | - | + | ? | - | High |
| Risperidone |  |  |  |  |  |  |  |  |  |  |
| Bobo et al., 2011 | + | - | - | - | - | + | ? | + | - | High |
| Antidepressants |  |  |  |  |  |  |  |  |  |  |
| Ghaemi et al., 2021 | + | + | - | + | + | + | + | + | + | Low |
| Parker et al., 2006 | ? | + | - | + | + | + | + | + | ? | Low |
| Post et al., 2006^a^ | ? | ? | + | + | + | + | - | ? | - | Moderate |
| Thyroid Hormones |  |  |  |  |  |  |  |  |  |  |
| Walshaw et al., 2018 | ? | ? | - | + | - | - | + | ? | + | Moderate |
| Omega-3 fatty acids |  |  |  |  |  |  |  |  |  |  |
| Keck et al., 2006 | + | + | + | + | + | + | + | ? | - | Low |
| Non-pharmacological |  |  |  |  |  |  |  |  |  |  |
| Lenz et al., 2016 | + | + | - | ? | - | + | + | ? | + | Moderate |

**Potential sources of bias: 1.** Allocation sequence random; **2.** Allocation adequately concealed; **3.**  Groups comparable at baseline; **4.** Blinding of group allocations; **5.** Groups treated equally; **6.** Intention-to-treat analysis^b^; **7.** Appropriate outcomes; **8.** Deviations from protocol; **9.** Allegiance effect minimized.

+ = Low Risk of Bias ? = Unclear Risk of Bias - = High Risk of Bias

^a^ Secondary analyses (See supplement 2 for additional detail)

^b^ *ITT approaches were coded as low risk of bias if analysing all participants randomised = ITT, or if a modified ITT (mITT) approach was taken but <5% of participants were excluded from analyses (due usually to no post-baseline data at all). An unclear risk of bias was coded if the study stated use of ITT or mITT but >5% of participants from the number randomised appeared not to have been analysed. A high risk of bias was scored where completer analysis was employed.*

*Overall risk was judged using the following criteria:*

Low risk = <2 criteria rated high RoB (or ≤2 if at least one is rated unclear RoB)

High risk = ≥4 criteria rated high RoB

Moderate risk = did not meet criteria for either high or low risk

**Supplement 5:** Additional efficacy indicators

| Reference | Intervention | N | | Global outcome | | Depression outcome | | Mania outcome | “Other” outcome | | Between Groups Summary | | | | Relapse / Remission / Response | | |
| --- | --- | --- | --- | --- | --- | --- | --- | --- | --- | --- | --- | --- | --- | --- | --- | --- | --- |
| Mood stabilisers | | |  | |  | |  | | |  |  |  |  |  | |  |  |
| Kemp et al., 2012 | Lamotrigine  Placebo | 23  26 | | CGI | | MADRS | | [YMRS] | n/a | | ~ | | | | 13% (depression remission)  31% (depression remission) | | |
| Wang et al., 2010 | Lamotrigine  Placebo | 18  18 | | CGI | | MADRS | | [YMRS] | n/a | | ~ | | | | 39% (depression response)  33% (depression response) | | |
| Suppes et al., 2008 ^a^ | Lamotrigine  Lithium | 35  36 | | CGI | | MADRS | | [YMRS] | n/a | | ~ | | | | 66% (depression remission)*  55% (depression remission)* | | |
| Calabrese et al., 2000 ^b^ | Lamotrigine  Placebo | 90  87 | | LIFE/CGI | | HAMD | | SADS | TTTC | | + lamotrigine euthymia | | | | 77% (remission) / 41% (sustain)  66% (remission) / 26% (sustain) | | |
| Goldsmith et al., 2003 | Lamotrigine  Placebo | 66  68 | | *CGI* | | *HAMD* | | [SADS] | TTTC | | ~ | | | | 25% (mania relapse) / 22% (MD relapse)  12% (mania relapse) / 46% (MD relapse) | | |
| Walden et al., 2000 | Lithium  Lamotrigine | 7  7 | | n/a | | HAMD | | YMRS | N relapse in 12m | | + lamotrigine relapse | | | | 43% (<4 relapses)  86% (<4 relapses) | | |
| Amsterdam et al., 2017 ^b^ | Venlafaxine  Lithium | 17  7 | | CGI | | HAMD | | [YMRS] | n/a | | ~ | | | | 59% (depression response)  39% (depression response) | | |
| Amsterdam et al., 2013 | Fluoxetine  Lithium  Placebo | 8  9  8 | | n/a | | HAMD | | YMRS | N relapse in 12m | | ~ | | | | 29% (depression relapse)  35% (depression relapse)  30% (depression relapse) | | |
| Amsterdam et al., 2009 | Venlafaxine  Lithium | 12  15 | | CGI | | HAMD | | [YMRS] | Time to (p)relapse | | + venlafaxine depression  ~ mania | | | | 75% (depression response)  27% (depression response) | | |
| Antipsychotics |  |  | |  | |  | |  |  | |  | | | |  | | |
| Tohen et al., 2006 | Olanzapine  Placebo | 119  60 | | n/a | | n/a | | n/a | TTR | | +olanzapine relapse | | | | 47% (relapse)*  80% (relapse)* | | |
| Suppes et al., 2005 ^b^ | Olanzapine  Divalproex | 76  68 | | CGI | | *HAMD* | | YMRS | n/a | | + olanzapine mania | | | | 57% (mania response)*  46% (mania response)* | | |
| Baldessarini et al., 2003 ^b^ | Olanzapine  Placebo | 44  46 | | CGI | | HAMD | | YMRS | TTI  N relapse in 12m | | + olanzapine global, mania | | | | 77% (mania response)  50% (mania response) | | |
| Tohen et al., 2003 | Olanzapine  Placebo  OFC | 132  127  34 | | CGI | | MADRS | | [YMRS] | TTI | | + OFC & olanzapine depression | | | | 33% (remission)*  25% (remission)*  49% (remission)* | | |
| Suppes et al., 2014 | Quetiapine  Placebo | 36  38 | | CGI | | MADRS | | [YMRS] | n/a | | + quetiapine global, depression | | | | 54% (remission)*  39% (remission)* | | |
| Cutler et al., 2011 | Quetiapine  Placebo | 45  52 | | CGI | | MADRS | | YMRS | n/a | | + Quetiapine global, mania, depression | | | | 42% (remission)*  28% (remission)* | | |
| Langosch et al., 2008 | Quetiapine  Sodium Valproate | 21  16 | | CGI | | *MADRS* | | [YMRS] | n/a | | + quetiapine global | | | | 43/48% (depression/mania response)  25/36% (depression/mania response) | | |
| McElroy et al. 2010 | Quetiapine  Paroxetine  Placebo | 81  24  24 | | CGI | | MADRS | | [YMRS] | n/a | | ~ | | | | 67% (remission)*  57% (remission)*  55% (remission)* | | |
| Vieta et al., 2007 ^b^ | Quetiapine  Placebo | 73  35 | | CGI | | MADRS | | [YMRS] | n/a | | +quetiapine global, depression | | | | 61/62% (BD-I/II response high dose)  77/57% (BD-I/II response low dose)  30/25% (BD-I/II response placebo) | | |
| Thase et al., 2006 | Quetiapine  Placebo | 90  53 | | CGI | | MADRS | | [YMRS] | n/a | | + quetiapine global, depression, mania | | | | 52% (remission)*  37% (remission)* | | |
| Muzina et al., 2008 ^b^ | Aripiprazole  Placebo | 14  14 | | n/a | | MADRS | | [YMRS] | TTR | | + aripiprazole TTR | | | | 14% (relapse)  43% (relapse) | | |
| Suppes et al., 2008b ^b^ | Aripiprazole  Placebo | 52  51 | | CGI | | MADRS | | YMRS | n/a | | + aripiprazole mania | | | | 48% (remission)  22% (remission) | | |
| Bobo et al., 2011 | Risperidone LAI  TAU | 20  25 | | CGI | | MADRS | | [YMRS] | N relapse in 12m | | ~ | | | | 60% necessary medication change 12m  96% necessary medication change 12m | | |
| Antidepressants | | | |  | |  | |  |  | |  | | | |  | | |
| Ghaemi et al., 2021 | Citalopram  Placebo | 14  19 | | CGI | | MADRS | | *SADS* | n/a | | ~ | | | | 34% (remission)*  34% (remission)* | | |
| Parker et al., 2006 | Escitalopram  Placebo | 10^c^ | | SOFAS | | HAMD | | YMRS | n/a | | + escitalopram global, depression, mania | | | | n=5 superior response SSRI vs placebo  n=4 no/minimal superiority of SSRI | | |
| Post et al., 2006 ^b^ | Bupropion  Sertraline  Venlafaxine | 21  12  14 | | CGI | | IDS | | [YMRS] | TTI/TTTC | | + bupropion switch | | | | 41% (remission)*  36% (remission)*  34% (remission)* | | |
| Other classes |  |  | |  | |  | |  |  | |  | | | |  | | |
| Walshaw et al., 2018 | T4  T3  Placebo | 13  10  9 | | n/a | | HAMD | | [YMRS] | n/a | | + levothyroxine vs placebo, euthymia | | | | NR | | |
| Keck et al., 2006 | Ethyl-EPA  Placebo | 31  28 | | CGI | | IDS | | YMRS | n/a | | ~ | | | | 25% mania relapse*  19% mania relapse* | | |
| Lenz et al., 2016 | CPT  Bibliotherapy | 7  9 | | CGI | | BRMES | | BRMAS | n/a | | ~ | | | | NR | | |

Outcomes in square brackets indicate unsuitability for meta-analysis due to lack of episode/substantial symptoms at baseline [average score not above standard ‘mild’ threshold’]. Outcomes in grey text indicate lack of sufficient data reported to indicate efficacy. Italics represent subsyndromal / removal in sensitivity analyses.

^a^ Data extracted from the whole study sample as there were no reported clinical differences between patients with or without RC

^b^ Secondary analyses (See supplement 2 for additional detail)

^c^ Reports data across all treatment groups

*Describes data regarding the overall study group as separate results for subjects with RC were not reported

CPT = cognitive psychoeducational therapy; RC = rapid cycling; NRC = non-rapid cycling; NA = not applicable; SSRI = selective serotonin reuptake inhibitors; NR = not reported; TTR = time to relapse; M = mean; SD = standard deviation; EI = early improvement; LTI =long-term improvement; MADRS = Montgomery-Asperg depression rating scale; YMRS = Young mania rating scale; HAMD = Hamilton depression rating scale; CGI = Clinical global impression; CGI-S = Clinical global impression-severity; CGI-M = Clinical global impression-mania; SADS = Mania rating scale from the schedule for affective disorders and schizophrenia; SOFAS =Social and Occupational Functioning Assessment Scale; TTTC = time to treatment change; BD-I = bipolar disorder I; BD-II = bipolar disorder II; PANSS-CS = Positive and negative Symptom scale cognitive score; CGI-BP = Clinical global impression scale for bipolar disorder; IDS = Inventory of Depressive Symptoms; LIFE = Prospective life chart method; n = number of patients, BRMES = Bech-Rafaelson Melancholia Scale, BRMAS = Bech-Rafaelson Mania Scale.

Within/between Groups Summary: (+) = improvement; (-) = worsening; (~) = no change

Difference between RC and Non-RC: (+) = improvement in subjects with RC/non-RC; (-) = worsening in subjects with RC/non-RC (~) = no difference between subjects with RC and non-RC.

**Supplement 6:** Log and rationale for sensitivity analyses

| Reference | Intervention | Outcomes examined | Inclusive of NRC | High RoB | Duration | Severity considerations | Sensitivity analysis decision |
| --- | --- | --- | --- | --- | --- | --- | --- |
| Kemp et al., 2012 | Lamotrigine / Placebo | CGI & MADRS | - all RC | - | - | - | N |
| Wang et al., 2010 | Lamotrigine / Placebo | CGI & MADRS | - all RC | - | - | - | N |
| Suppes et al., 2008 | Lamotrigine / Lithium | CGI & MADRS | NRC included but NS difference | - | - | **-** | N |
| Goldsmith et al., 2003 | **Lamotrigine / Placebo** | **CGI & HAMD** | **- all RC** | **-** | **-** | **Partial Remission** | **Y – CGI & HAMD (both arms)** |
| Amsterdam et al., 2009 | Venlafaxine / Lithium | HAMD & TTR | - only RC analysed | - | - | - | N |
| Suppes et al., 2005 | Olanzapine / Divalproex | CGI & HAMD & YMRS | NRC potential effect for HAMD / YMRS but only RC analysed in CGI | - | - | **-** | N |
| Baldessarini et al., 2003 | **Olanzapine / Placebo** | **YMRS** | **- only RC analysed** | **High** | **Low** | **-** | **Y – all** |
| Tohen et al., 2003 | **Olanzapine / Placebo / OFC** | **CGI & MADRS** | **NRC potential effect for CGI, but only RC analysed in MADRS** | **-** | **-** | **-** | **Y – CGI only (both arms)** |
| Suppes et al., 2014 | Quetiapine / Placebo | CGI & MADRS | NRC included but NS difference | - | - | **-** | N |
| Cutler et al., 2011 | **Quetiapine / Placebo** | **CGI & MADRS & YMRS** | **NRC potential effect for MADRS / CGI but only RC analysed in YMRS** | **-** | **Low** | **Low depression symptoms** | **Y – placebo (CGI & MADRS)** |
| Langosch et al., 2008 | **Quetiapine / Sodium Valproate** | **MADRS** | **- all RC** | **High** | **High** | **Partial Remission** | **Y – all outcomes** |
| McElroy et al. 2010 | Quetiapine / Paroxetine / Placebo | MADRS | - only RC analysed | - | - | - | N |
| Vieta et al., 2007 | Quetiapine / Placebo | CGI & MADRS | - only RC analysed | - | **-** | - | N |
| Muzina et al., 2008 | **Aripiprazole / Placebo** | **MADRS** | **- all RC** | **-** | **High** | **Low depression symptoms** | **Y – placebo MADRS** |
| Thase et al., 2006 | Quetiapine / Placebo | MADRS & CGI | NRC incl. (CGI) but NS difference; only RC analysed in MADRS | - | - | **-** | N |
| Suppes et al., 2008b | Aripiprazole / Placebo | YMRS | - only RC analysed | High | Low | **-** | N |
| Bobo et al., 2011 | **Risperidone LAI / TAU** | **CGI & MADRS** | **- all RC** | **High** | **High** | **-** | **Y – placebo CGI** |
| Ghaemi et al., 2021 | Citalopram / Placebo | CGI & MADRS & SADS | NRC potential effect for CGI / MADRS; only RC analysed in SADS | - | High | - | N |
| Parker et al., 2006 | Escitalopram / Placebo | SOFAS & HAMD & YMRS | - all RC | - | - | Particularly severe RC | N |
| Walshaw et al., 2018 | T4 / T3 / Placebo | HAMD | - all RC | - | **-** | - | N |
| Lenz et al., 2016 | CPT / Bibliotherapy | CGI | - only RC analysed | - | - | Partial Remission | N |

Rows in bold represent those excluded due to inclusion increasing heterogeneity between studies within (any) analysis.

The following were excluded from all analyses and from this table due to lack of continuous outcome data: Keck et al., 2006; Post et al., 2006; Amsterdam et al., 2013 and 2017

**Supplement 7:** Additional relevant efficacy outcomes not suitable for inclusion in meta-analyses

| Outcome | Reference | Intervention | Data | Duration covered | Methodological considerations |
| --- | --- | --- | --- | --- | --- |
| Time to treatment change | Calabrese et al., 2000 | Lamotrigine / Placebo | Median survival 18 weeks lamotrigine; 12 weeks placebo | 26 Weeks | Partial remission at baseline |
|  | Goldsmith et al., 2003 | Lamotrigine / Placebo | Mean (SD) = 142 (127) vs 133 (77) days | 32 Weeks | Partial remission at baseline |
| Time to relapse | Amsterdam et al., 2009 | Venlafaxine / Lithium | Mean time to first increase in mania scores: 3 days venlafaxine; 12 days lithium | 12 weeks | Depressed at baseline |
|  | Tohen et al., 2006 | Olanzapine / Placebo | Mean = 174 vs 22 days | 48 Weeks | Euthymic at baseline |
|  | Muzina et al., 2008 | Aripiprazole / Placebo | Aripiprazole: At 101 days, 81% had not relapsed.  Placebo: mean time to relapse 118 (24.5) days | 100 weeks | Euthymic at baseline |
| N relapses | Walden et al., 2000 | Lithium / Lamotrigine | N relapses after response: 30 lithium, 11 lamotrigine (7 patients per arm) | 52 Weeks | Manic at baseline |
|  | Bobo et al., 2011 | Risperidone LAI / TAU | N (SD) = 5.34 (2.35) risperidone vs 6.26 (3.24) TAU | 52 Weeks | High ROB |
|  | Baldessarini et al., 2003 | Olanzapine | N (SD) = 2.98 (3.92) | 3/4weeks | High ROB |
| Time to improve | Baldessarini et al., 2003 | Olanzapine / Placebo | Mean (SD) 1 (0.13) vs 2 (0.16 weeks | 3/4 weeks | High ROB |

**Supplement 8:** Effects of treatment duration on meta-analysis outcomes

| Outcome | Treatment | < 6 weeks | | | | | 6-52 weeks | | | | | > 52 weeks | | | | |
| --- | --- | --- | --- | --- | --- | --- | --- | --- | --- | --- | --- | --- | --- | --- | --- | --- |
|  |  | k | N | ES | 95% CI | I^2^ | k | N | ES | 95% CI | I^2^ | k | N | ES | 95% CI | I^2^ |
| *Global Impression* | **Antipsychotics** | **1** | **159** | **0.73** | **0.56 – 0.91** | **-** | **4** | **721** | **0.81** | **0.72 – 0.89** | **0%** | **1** | **20** | **0.56** | **0.08 – 1.03** | **-** |
|  | Quetiapine | [see above row – antipsychotics global] | | | | | 3 | 645 | 0.79 | 0.68 – 0.90 | 12% | - | - | - | - | - |
| *Depression* | **Antipsychotics** | **1** | **159** | **0.65** | **0.48 – 0.82** | **-** | **7** | **938** | **0.90** | **0.75 – 1.05** | **37%** | **2** | **34** | **0.24** | **-0.10 – 0.58** | **0%** |
|  | Quetiapine | [see above row – antipsychotics depression] | | | | | 4 | 696 | 0.81 | 0.60 – 1.03 | 34% | - | - | - | - | - |
| *Mania* | **Antipsychotics** | **2** | **211** | **1.05** | **0.80 – 1.30** | **0%** | **1** | **76** | **1.19** | **0.90 – 1.49** | **-** | **-** | **-** | **-** | **-** | **-** |
| *Depression* | **Antidepressants** | **-** | **-** | **-** | **-** | **-** | **3** | **42** | **1.37** | **0.50 – 2.25** | **68%** | **1** | **59** | **1.10** | **0.78 – 1.43** | **-** |
| *Global Impression* | **Controls** | **1** | **149** | **1.14** | **0.94 – 1.35** | **-** | **6** | **288** | **0.64** | **0.51 – 0.77** | **0%** | **2** | **84** | **0.50** | **-0.77-1.17** | **87%** |
|  | Placebo | [see above row – controls global] | | | | | [see above row – controls global] | | | | | 1 | 59 | 0.83 | 0.53-1.12 | - |
| *Depression* | **Controls** | **1** | **149** | **0.97** | **0.77 – 1.17** | **-** | **9** | **437** | **0.57** | **0.37 – 0.76** | **56%** | **3** | **98** | **0.54** | **0.13 – 0.94** | **67%** |
|  | Placebo | [see above row – controls depression] | | | | | [see above row – controls depression] | | | | | 2 | 73 | 0.50 | -0.23 – 1.22 | 83% |
| *Mania* | Placebo | 2 | 103 | 0.38 | 0.15 – 0.61 | 23% | 1 | 4 | 0.27 | -0.73 – 1.26 | - | 1 | 19 | 0.08 | -0.37 – 0.53 | - |

Other outcomes with >3 studies were all of the same duration (all mood stabilisers global and depression outcomes were 6-52 weeks).
